# *Mycobacterium intracellulare* induces a Th17 immune response via M1-like macrophage polarization in canine peripheral blood mononuclear cells

**DOI:** 10.1038/s41598-022-16117-2

**Published:** 2022-07-12

**Authors:** Suji Kim, You-Seok Hyun, Hong-Tae Park, Min-Kyung Shin, Han Sang Yoo

**Affiliations:** 1 Department of Infectious Diseases, College of Veterinary Medicine, Seoul National University, Seoul, Republic of Korea; 2 BK21 FOUR Future Veterinary Medicine Leading Education and Research Center, Seoul National University, Seoul, Republic of Korea; 3 Department of Microbiology, College of Medicine, The Catholic University of Korea, Seoul, Republic of Korea; 4 Department of Microbiology, College of Medicine, Gyeongsang National University, Jinju, Republic of Korea; 5 Bio-MAX/N-Bio Institute, Seoul National University, Seoul, Republic of Korea

**Keywords:** Bacterial infection, Microbiology, Bacteria, Bacterial immune evasion

## Abstract

*Mycobacterium avium*-*intracellulare* complex (MAC) is one of the most prevalent pathogenic nontuberculous mycobacteria that cause chronic pulmonary disease. The prevalence of MAC infection has been rising globally in a wide range of hosts, including companion animals. MAC infection has been reported in dogs; however, little is known about interaction between MAC and dogs, especially in immune response. In this study, we investigated the host immune response driven by *M. intracellulare* using the co-culture system of canine T helper cells and autologous monocyte-derived macrophages (MDMs). Transcriptomic analysis revealed that canine MDMs differentiated into M1-like macrophages after *M. intracellulare* infection and the macrophages secreted molecules that induced Th1/Th17 cell polarization. Furthermore, canine lymphocytes co-cultured with *M. intracellulare*-infected macrophages induced the adaptive Th17 responses after 5 days. Taken together, our results indicate that *M. intracellulare* elicits a Th17 response through macrophage activation in this system. Those findings might help the understanding of the canine immune response to MAC infection and diminishing the potential zoonotic risk in One Health aspect.

## Introduction

Nontuberculous mycobacteria (NTM) are opportunistic bacteria that are highly abundant in environment niches such as soil and water sources. *Mycobacterium avium*-*intracellulare* complex (MAC) is one of the most prevalent pathogenic NTM that causes chronic pulmonary diseases in humans and other mammals. In dogs, MAC infection can cause mycobacterial granulomas in various organs, including the spleen, liver, lung, bone marrow, and various lymph nodes^1,2^. Dogs with mycobacteriosis caused by MAC usually have poor prognoses due to the difficulty of diagnosis and nonspecific clinical signs. Although diagnosed, most affected dogs die or are euthanized regardless of any treatment after the progress of the infection. A recent study reported that domestic dogs are more susceptible to MAC infection because of their restricted genetic pool^3^. Furthermore, there is a potential zoonotic risk of transmission from infected companion dogs to their owners, especially in immunocompromised people, although interspecies transmission of MAC is still remaining to be clear. Some reports have shown that MAC members can be transmitted from dogs to humans^4^. In addition, NTM infection mostly occurs by exposure to environmental sources because of the ubiquitous nature. Our previous study showed that MAC accounted for most of NTM isolated from soil that collected in animal shelter and parks^5^. Therefore, it is important to understand the pathogenicity of MAC in dogs not only for treatment and diagnosis but also to prevent potential zoonotic transmission between humans and dogs.

Macrophages are considered to be a primary reservoir for intracellular mycobacterial growth and to be important antigen-presenting cells^6^. Macrophage plasticity is key for mycobacterial control, and macrophages polarize to the classical (M1) or alternative (M2) phenotypes in response to bacterial infection^7^. M1 macrophages release high levels of proinflammatory cytokines that have enhanced microbicidal activity, while M2 macrophages produce anti-inflammatory cytokines and cause persistent infection^8,9^. Macrophages have been reported to exhibit transformation from the M1 to the M2 phenotype over the course of mycobacterial infection^7,10^. However, recent studies have shown that *Mycobacterium*-infected macrophages polarize to a unique macrophage population that is clearly distinguishable from the M1 and M2 macrophage subsets^11^. In particular, *M. intracellulare*-infected macrophages represent a novel polarized population that downregulates Th1/Th2 cell production while driving Th17 polarization^12^.

In mycobacterial infection, CD4^+^ T cells are the most dominant in the immune response^13^. The Th1/Th17 balance is thought to be important for protection against mycobacteria^14,15^. The Th1 immune response is essential for controlling mycobacterial infection. Th17 cells participate in the antimycobacterial response by accelerating the accumulation of Th1 cells at an early stage^16,17^. However, the Th17 response has pathological effects during MAC infection under Th1-diminished conditions. Th17 development induces excessive neutrophilic pulmonary inflammation by reducing Th1 responses and can increase susceptibility to systemic MAC infection^15^. Inflammation and neutrophil recruitment driven by Th17 cells play a pivotal role in granuloma formation and mycobacterial disease development^18,19^.

As mycobacteriosis caused by MAC in dogs has been consistently increased, host immune response should be revealed to understand the pathogenesis in MAC-infected dogs. As the first step, an in vitro canine model was developed to investigate the immune response to *M. intracellulare* infection. Transcriptomic analysis in canine monocyte-derived macrophages was carried out to identify activated macrophages driven by the bacterial infection. Also, the T cell response was investigated using *M. intracellulare*-infected autologous macrophages. These results will help further our understanding of the pathogenesis of *M. intracellulare*-related crosstalk between macrophages and T helper cells. Furthermore, this study helps to establish a basis for research on MAC infections, which occur in a wide range of hosts, as well as for understanding the mechanism of *M. intracellulare* infection in dogs.

## Results

### Transcriptome analysis of canine monocyte-derived macrophages against *M. intracellulare* infection

To identify the canine immune response against *M. intracellulare*, canine MDMs were analyzed through RNA-Seq after infection with *M. intracellulare* for 0, 6, 24, and 72 h. Sixteen cDNA libraries from noninfected and *M. intracellulare*-infected cells were sequenced. A total of 1542 differentially expressed genes (DEGs) were significantly expressed in the *M. intracellulare* infection group compared to the uninfected group. Transcriptional profiles were analyzed by Ingenuity Pathway Analysis (IPA) using the significant DEGs ($| {fold\, change} | \ge 1.5$). A comparison analysis was performed using canonical pathways that were expressed at each time point (6 h, 24 h, 72 h). Table 1 shows the pathway commonly expressed during *M. intracellulare* infection. The top 20 pathways were related to macrophage activation and induction of T helper cell responses. The activated pathways (Role of IL-17F in Allergic Inflammatory Airway Diseases, IL-6 Signaling, TREM1 Signaling, HMGB1 Signaling, Th17 Activation Pathway, Role of Pattern Recognition Receptors in Recognition of Bacteria and Viruses, CD28 Signaling in T Helper Cells, CD40 Signaling, IL-23 Signaling Pathway, Dendritic Cell Maturation) were involved in M1 macrophage activation. In contrast, inactivated pathways (LXR/RXR Activation, PPARα/RXRα Activation, PPAR Signaling) were related to M2 macrophage differentiation. The activation states were predicted by particular genes expressed in those pathways. Among the genes, the upregulated genes (*CCL3*, *CCL4*, *CCL5*, *IL6*, *IL1B*, *ICAM1*, *CSF2, TREM1*, *CD40*) were related to M1 macrophage activation, and the downregulated gene *CD36* was related to M2 macrophages (Supplementary Table S1).

**Table 1:** Top 20 canonical pathways in *M. intracellulare*-infected canine MDMs as determined by comparison analysis of IPA. The canonical pathways are indicated by the z-score determined from the pathway activation analysis.

Canonical pathways	6 h	24 h	72 h
Role of IL-17F in allergic inflammatory airway diseases	3	2.714	2.333
IL-6 signaling	2.84	1.807	2.668
TREM1 signaling	3.578	2.828	0.688
HMGB1 signaling	2.524	2	2.183
LXR/RXR activation	− 1.789	− 2.673	− 2.183
Th17 activation pathway	2.236	1.633	2.333
Hepatic fibrosis signaling pathway	3.087	0.169	2.412
FAT10 cancer signaling pathway	2.449	0.816	2.236
PPARα/RXRα activation	− 1.897	− 1.265	− 2.309
Renin–angiotensin signaling	2.121	1.667	1.667
Cholecystokinin/gastrin-mediated signaling	2.53	1.667	1.155
Role of pattern recognition receptors in recognition of bacteria and viruses	2.887	1.732	0.535
PPAR signaling	− 1.155	− 2.333	− 1.604
CD28 signaling in T helper cells	1.633	1.633	1.667
MIF-mediated glucocorticoid regulation	2.236	1.342	1.342
CD40 signaling	2	1.134	1.633
Osteoarthritis pathway	2.357	1.414	0.943
Cardiac hypertrophy signaling (enhanced)	1.512	1.633	1.567
IL-23 signaling pathway	2	0.447	2.236
Dendritic cell maturation	3.838	0	0.816

The pathways were also involved in the induction of the T cell response. The activated pathways TREM1 Signaling, HMGB1 Signaling and Dendritic Cell Maturation were associated with the Th1 response, and other activated pathways (Role of Th17F in Allergic Inflammatory Airway Diseases, IL-6 Signaling, Th17 Activation Pathways, IL-23 Signaling Pathway) were related to the Th17 response. In addition, the inactivated pathways (LXR/RXR Activation, PPARα/RXRα Activation, PPAR Signaling) were involved in the inhibition of the Th17 response. Of the molecules expressed in these pathways, Th1/Th17 cell response-related molecules (*CCL3*, *CCL4*, *CCL5*, *CCL7*, *IL1B*, *IL6*, *CSF2*, *CXCL8*, *ICAM1*, *S100A8*, *PTGS2*, *CD40*, *CD80*) were commonly upregulated during *M. intracellulare* infection. However, *IL15*, which is known to be related to the Th1 cell response, was inactivated (Supplementary Table S1).

### Canine macrophages differentiate into M1-like macrophages after *M. intracellulare* infection

**Figure 1:**
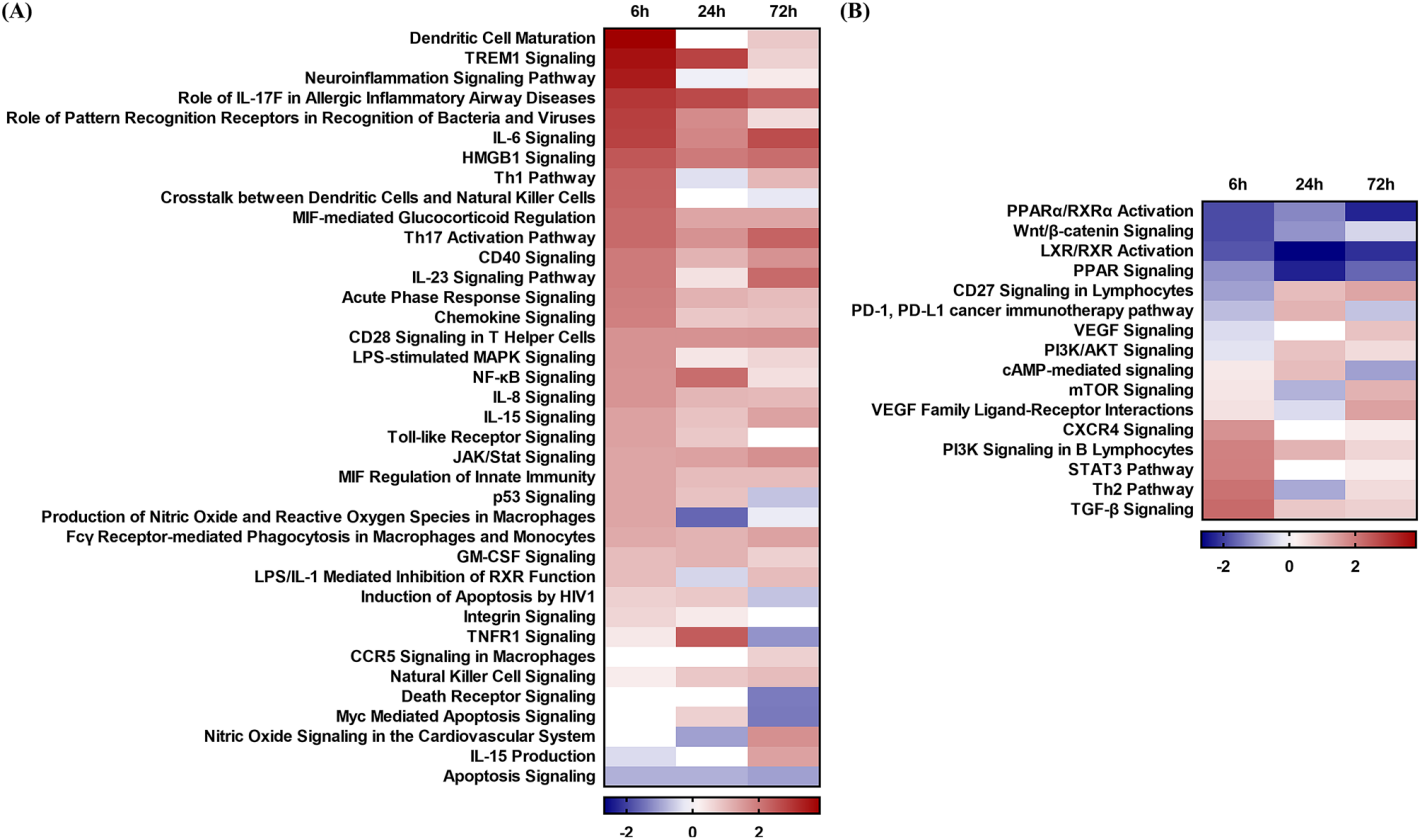
Heatmap of the canonical pathways related to macrophage activation in canine MDMs infected with *M. intracellulare*. The heatmap displays the canonical pathways related to (**A**) M1 macrophage and (**B**) M2 macrophage polarization. The color gradient reflects the predicted directions based on the z-score, where blue represents inhibition and red represents activation.

After identifying the commonly expressed pathways, at 6 h, 24 h, and 72 h, each pathway was analyzed in relation to macrophage activation. Pathways were trimmed based on significance $[ { - log ( {p{\text{-}}value} ) \ge 1.3} ]$ (Supplementary Table S2, Supplementary Table S3, Supplementary Table S4). The activation status was predicted by the z-score. Figure 1A shows that most M1 macrophage-associated pathways were activated, whereas Fig. 1B shows that M2 macrophage-associated pathways were negatively regulated or inactivated. The grey boxes in Supplementary Table S5 indicate pathways that were continuously activated or inactivated throughout *M. intracellulare* infection. However, some pathways were not consistent with the M1 activation states. In particular, apoptosis-related pathways (Apoptosis Signaling, Myc Mediated Apoptosis Signaling, Induction of Apoptosis by HIV1, Production of Nitric Oxide and Reactive Oxygen Species in Macrophages) were negatively regulated over time. The Apoptosis Signaling pathway was predicted to be inactivated throughout the infection.

**Figure 2:**
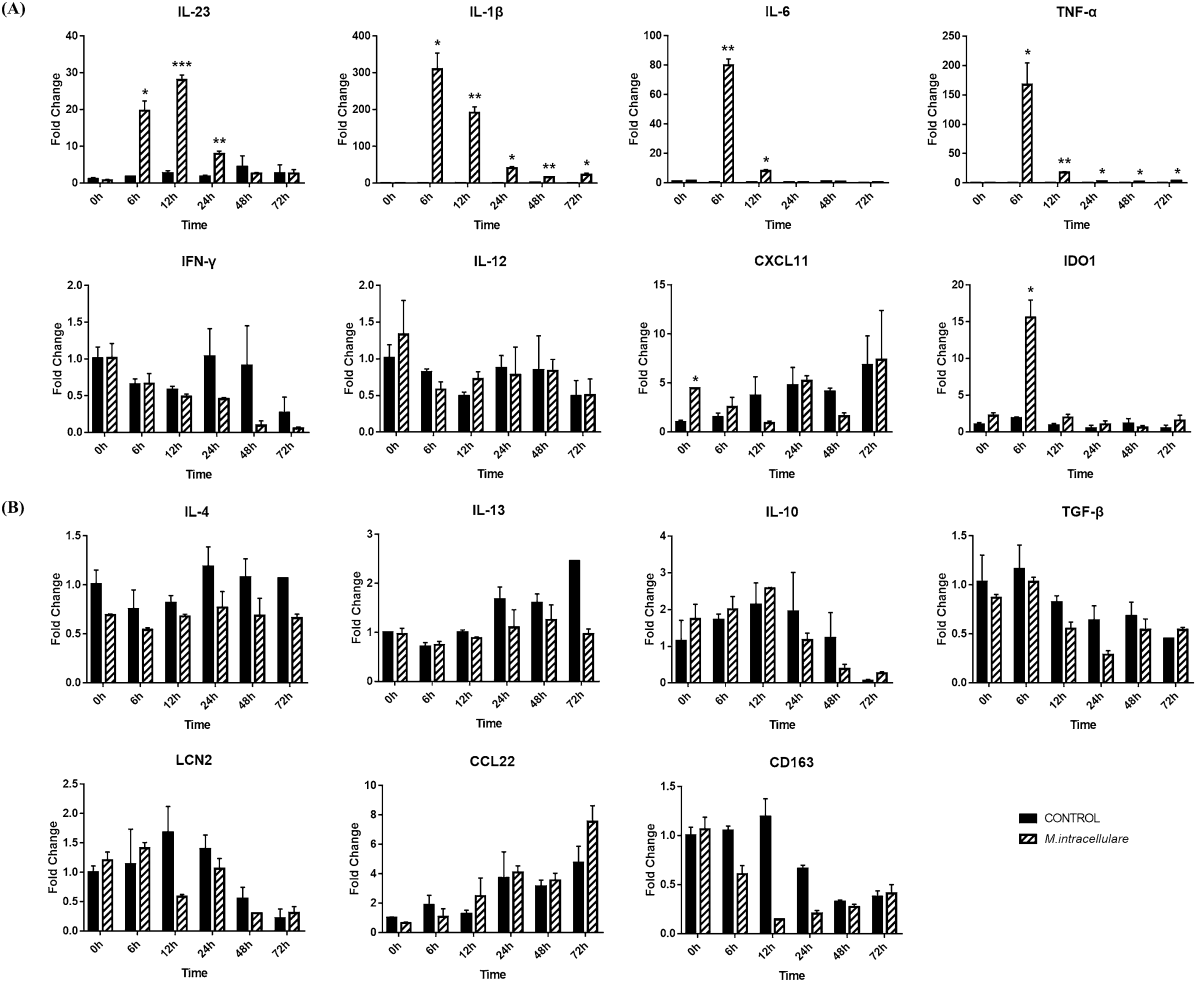
Gene expression analysis of canine MDMs infected with *M. intracellulare*. The expression level of genes related to (**A**) M1 and (**B**) M2 macrophages is presented as fold-change in mRNA expression. The mRNA expression was analyzed chronologically after canine MDMs were infected with *M. intracellulare* for 0–72 h. The mRNA expression in noninfected cells at 0 h was given a value of 1 as a reference for fold-change in expression. Each bar represents the mean ± SEM from three independent experiments in individual dogs. *p < 0.05, **p < 0.01, and ***p < 0.001.

Because of this discrepancy with the characteristics of classical M1 macrophages, macrophage activation-related genes were quantified by RT-qPCR during 72 h of infection. The expression levels of M1 macrophage markers (*IL-23*, *IL-1β*, *IL-6*, *TNF-α*, *IDO1*) were significantly upregulated in *M. intracellulare*-infected MDMs compared to noninfected MDMs (Fig. 2A). In contrast, M2 macrophage markers did not show any significant changes during *M. intracellulare* infection (Fig. 2B). An invasion assay also showed that *M. intracellulare* replicated in canine MDMs after infection. The amount of intracellular *M. intracellulare* increased after 5 days of infection (Supplementary Fig. S1). These results indicate that apoptosis signaling was inactivated despite the M1 markers being upregulated during the infection.

### *M. intracellulare*-infected canine macrophages produce molecules to induce the T helper cell response

**Figure 3:**
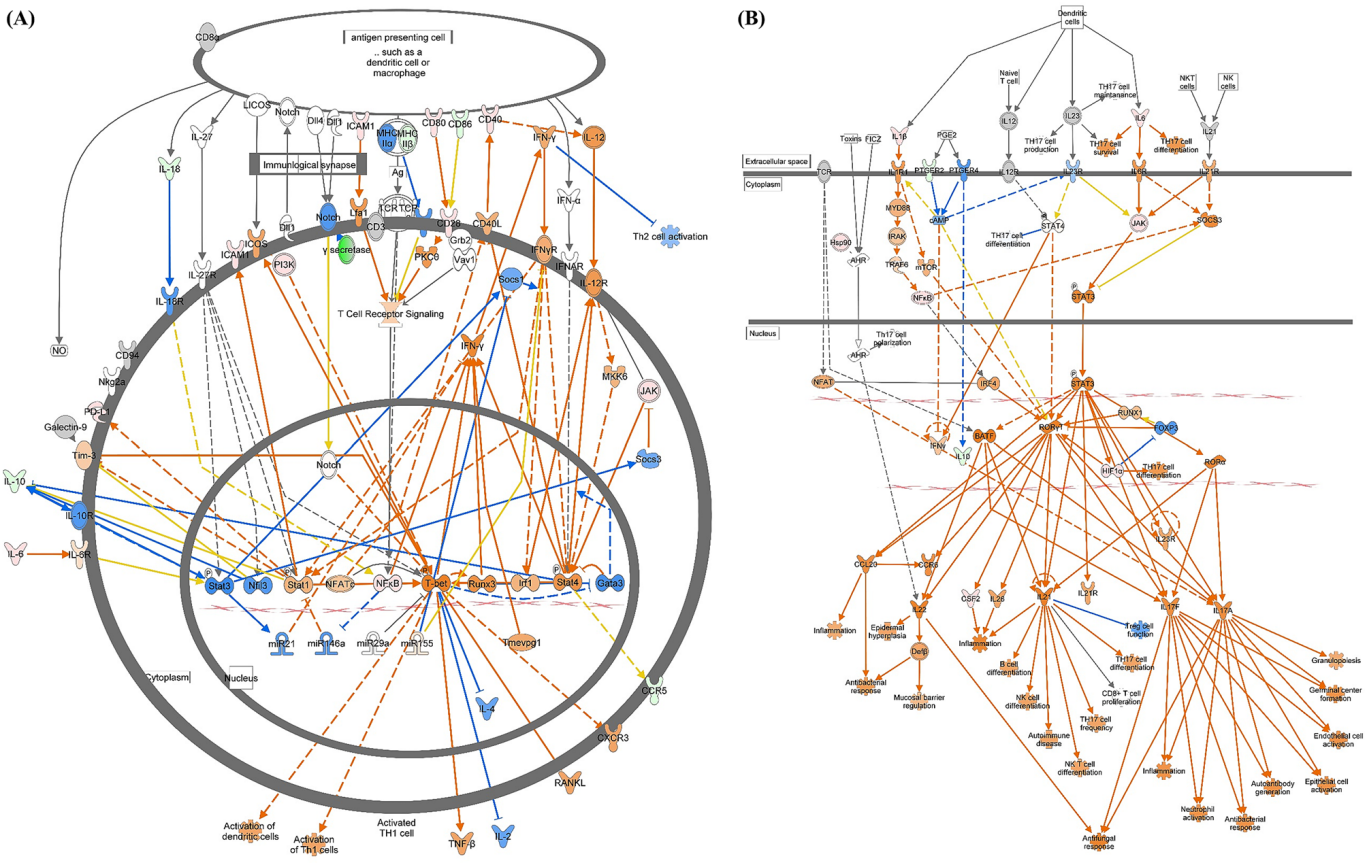
Ingenuity pathway analysis of the T helper cell response in *M. intracellulare*-infected canine MDMs. (**A**) The Th1 Pathway and (**B**) Th17 Activation Pathway were expressed at 72 h post infection. The individual nodes represent proteins with relationships represented by edges. The red and green colors indicate up- and downregulation based on the expression values, respectively. The activation states by z-scores are shown as follows: orange indicates activation, blue indicates inactivation, and uncolored nodes indicate genes that were not differentially expressed in this pathway.

Analysis of pathways commonly activated during infection showed that macrophages induce Th1 and Th17 responses upon *M. intracellulare* infection. Most of the molecules upregulated in that pathway were also associated with both Th1 and Th17 responses, although some molecules involved in the Th1 response were downregulated. To specifically identify the expression of genes that induce Th1/Th17 cell responses, the activation states of the Th1 Pathway and Th17 Activation Pathway were analyzed. The Th1 Pathway was activated at only 6 and 72 h $( {z{\text{-}} score;\,\, 6\,h = 2.333,\,\, 24\,h = - 0.333,\,\, 72\,h = 1.069} )$ by upregulated genes (*CD40*, *CD80*, *ICAM1*, *IL-6*, *NF-κB*) (Fig. 3A, Supplementary Table S2, Supplementary Table S3, Supplementary Table S4). On the other hand, the Th17 Activation Pathway was activated throughout *M. intracellulare* infection $( {z{\text{-}} score;\,\,6\,h = 2.236,\,\, 24\,h = 1.633,\,\, 72\,h = 2.333} )$ because Th17-associated molecules (*CSF2*, *IL-1B*, *IL-6*) were upregulated (Fig. 3B, Supplementary Table S2, Supplementary Table S3, Supplementary Table S4). These results were also confirmed by analysis of the expression of particular genes by RT-qPCR. Gene expression profiling revealed that the expression of the *IL-23*, *IL-1β*, and *IL-6* genes, which induce Th17 cell polarization, was significantly increased compared to that in the control group. Among Th1-related genes (*IFN-γ*, *TNF-α*, *IL-12*), only TNF-α showed significantly increased expression. Th2 (*IL-4*, *IL-13*) and Treg (*IL-10*, *TGF-β*)-related genes did not show significantly increased expression in canine MDMs infected with *M. intracellulare* (Fig. 2).

### Th17 cell response in canine lymphocytes co-cultured with MDMs after *M. intracellulare* infection

**Figure 4:**
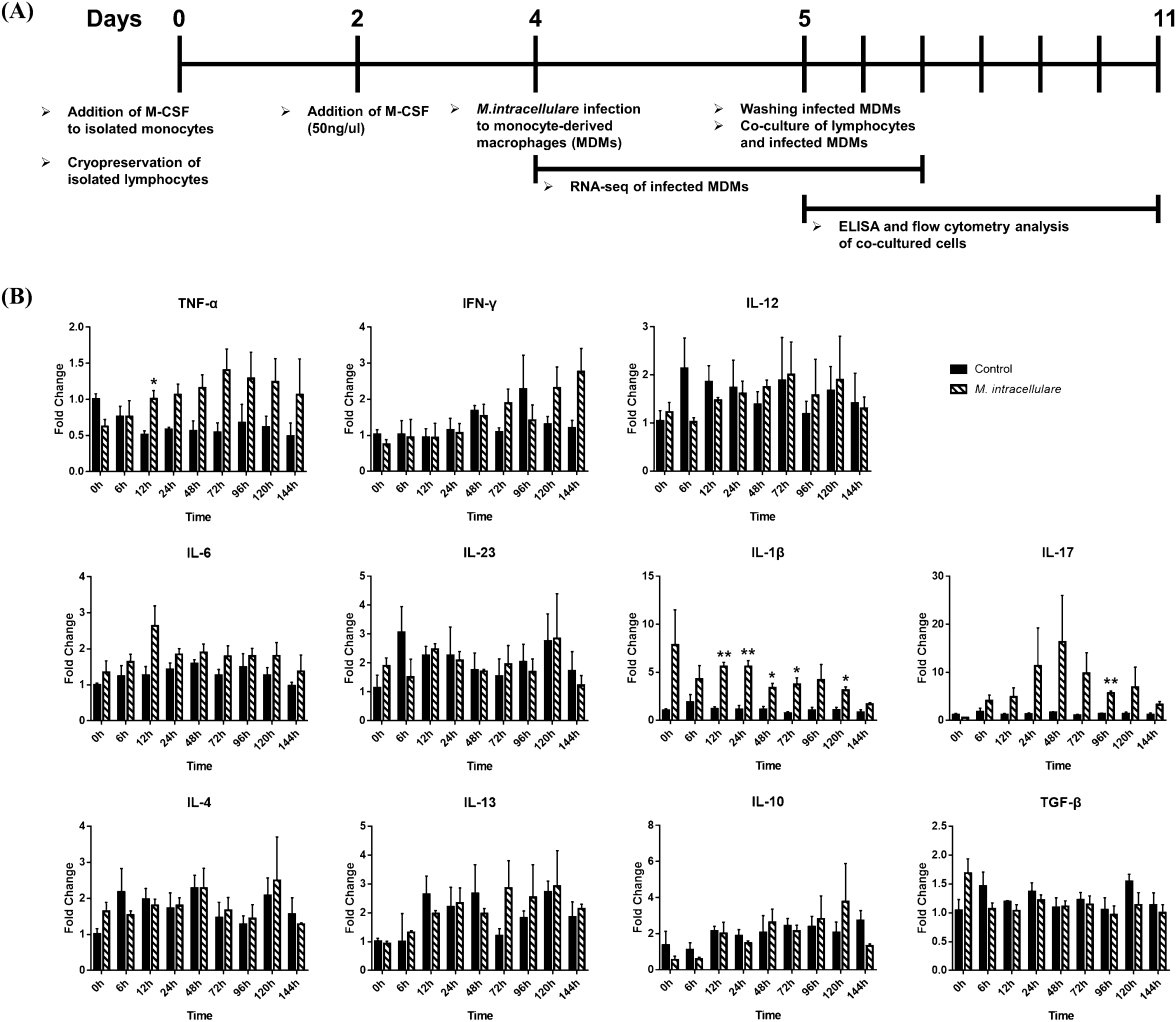
Differential cytokine expression in mRNA level of monocyte-depleted PBMCs co-cultured with *M. intracellulare*-infected canine MDMs. (**A**) Flowchart of co-culture experiment for the T helper cell response. (**B**) The cytokines TNF-α, IFN-γ, and IL-12 (Th1); IL-4 and IL-13 (Th2); IL-6, IL-23, IL-1β, and IL-17 (Th17); and IL-10 and TGF-β (Treg) were quantified based on their fold changes of the mRNA expression in canine monocyte-depleted PBMCs co-cultured with *M. intracellulare*-infected MDMs. The mRNA expression in non-infected cells at 0 h was given a value of 1 as a reference for fold change in expression. Each bar represents the mean ± SEM from three independent experiments in individual dogs. *p < 0.05, **p < 0.01.

**Figure 5:**
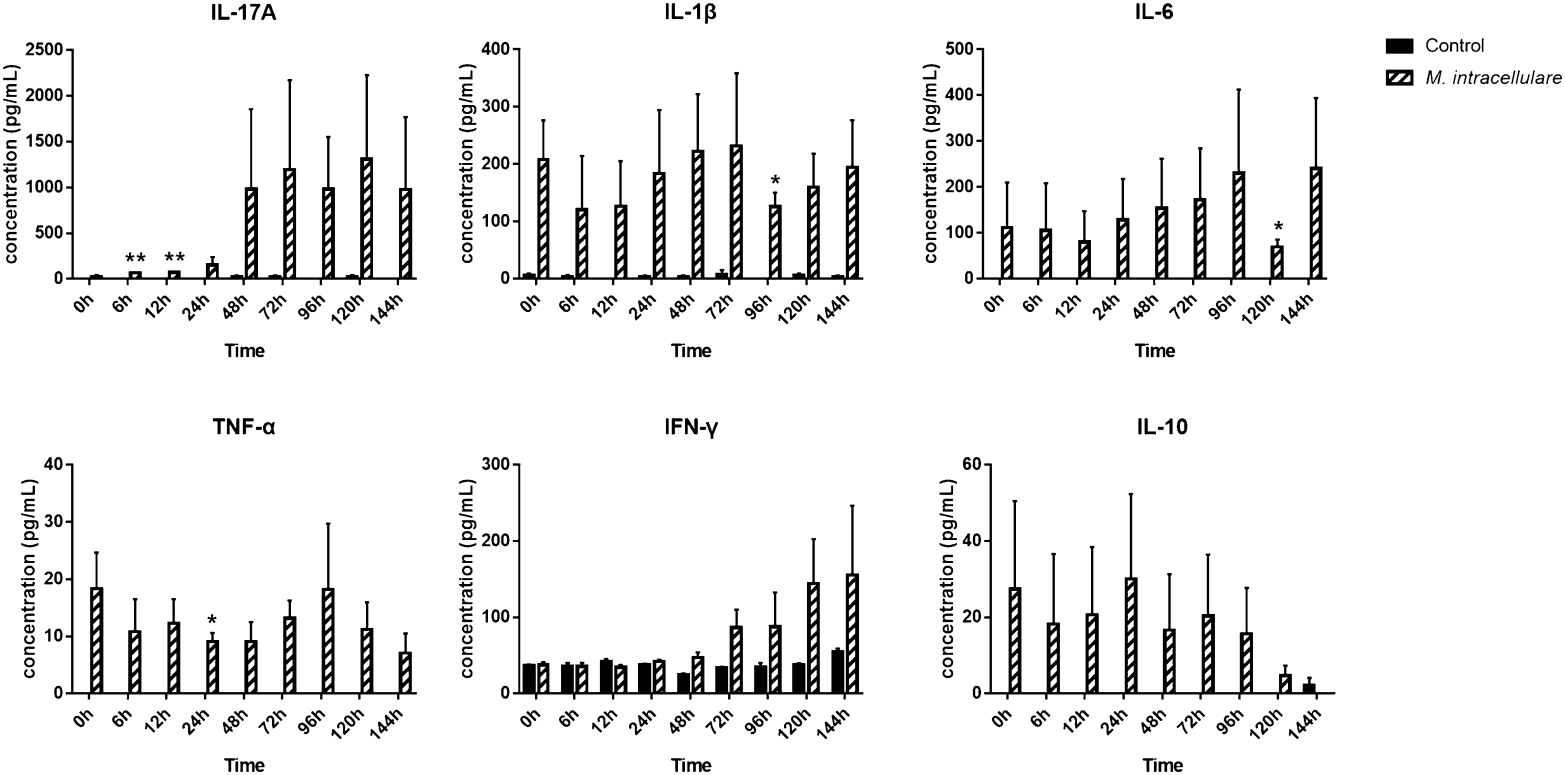
Cytokine expression in canine monocyte-depleted PBMCs co-cultured with canine MDMs after *M. intracellulare* infection. The concentration of IL-17A, IL-1β, IL-6, TNF-α, IL-12, IFN-γ, IL-10, and IL-4 was determined by ELISA. IL-4 and IL-12 were not detected. Cytokine expression in noninfected cells at each time point was used as a control. Each bar represents the mean ± SEM from three independent experiments in individual dogs. *p < 0.05, **p < 0.01.

Lymphocytes were co-cultured for 6 days with autologous MDMs infected for 24 h with *M. intracellulare* to investigate the T cell response (Fig. 4A). T cells were enriched mainly after 5 days of co-culture (Fig. 7A, Supplementary Fig. S2). Gene expression profiling of co-cultured lymphocytes showed that Th17-related genes (*IL-1β*, *IL-17*) were significantly upregulated compared to the control (Fig. 4B). In particular, the gene expression of IL-17 was highest among cytokines associated with subsets of CD4^+^ helper T cells. Other cytokines associated with Th1 (*IFN-γ*, *IL-12*), Th2 (*IL-4*, *IL-13*), and Tregs (*IL-10*, *TGF-β*) were not significantly upregulated except for *TNF-α*. However, *TNF-α* was significantly expressed only at 12 h, and the expression level was also very low $( {fold\, change = 1.007 \pm 0.117} )$. We then analyzed the supernatants of lymphocytes with ELISA to confirm the production of cytokines (Fig. 5). All cytokines related to subsets of CD4^+^ helper T cells were secreted upon *M. intracellulare* infection, except the cytokines related to Th2. Th17-related cytokines (IL-17A, IL-1β) expressed higher than other cytokines produced in Th1 cells (TNF-α, IFN-γ) and Tregs (IL-10).

**Figure 6:**
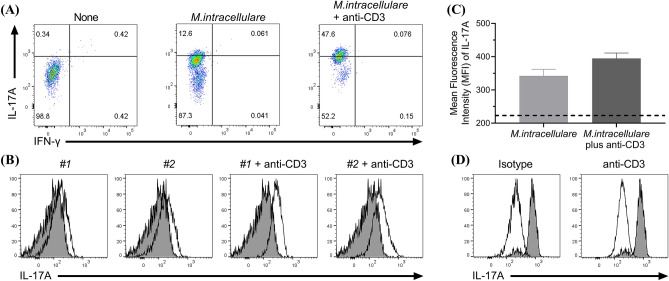
IL-17- and IFN-γ-producing CD4^+^ T cells according to *M. intracellulare* infection and anti-CD3 stimulation. (**A**) Representative frequency of IL-17- or IFN-γ- producing canine CD4^+^ T cells in untreated lymphocytes (left), co-cultured with *M. intracellulare*-infected non-autologous MDMs (middle) or co-cultured with *M. intracellulare*-infected non-autologous MDMs with the anti-CD3 antibody (right). (**B**) Canine CD4^+^ T cells were co-cultured with *M. intracellulare*-infected non-autologous MDMs (#1, #2) and activated with anti-CD3 antibody (#1 + anti-CD3, #2 + anti-CD3). IL-17-expressing CD4^+^ T cells was analyzed by comparing untreated lymphocytes (filled). (**C**) The mean fluorescence intensity of IL-17 in co-cultured CD4^+^ T cells with *M. intracellulare*-infected non-autologous MDMs without (*M. intracellulare*) or with anti-CD3 antibodies (*M. intracellulare* plus anti-CD3), respectively. A dotted line indicates the expression of IL-17 in untreated lymphocytes. (**D**) In co-cultured with *M. intracellulare*-infected non-autologous MDMs with the anti-CD3 antibody, IL-17-expressing CD4^+^ T cells was analyzed by comparing isotype control (left). The co-cultured cells with non-infected MDMs with anti-CD3 antibody were used as a control (right).

**Figure 7:**
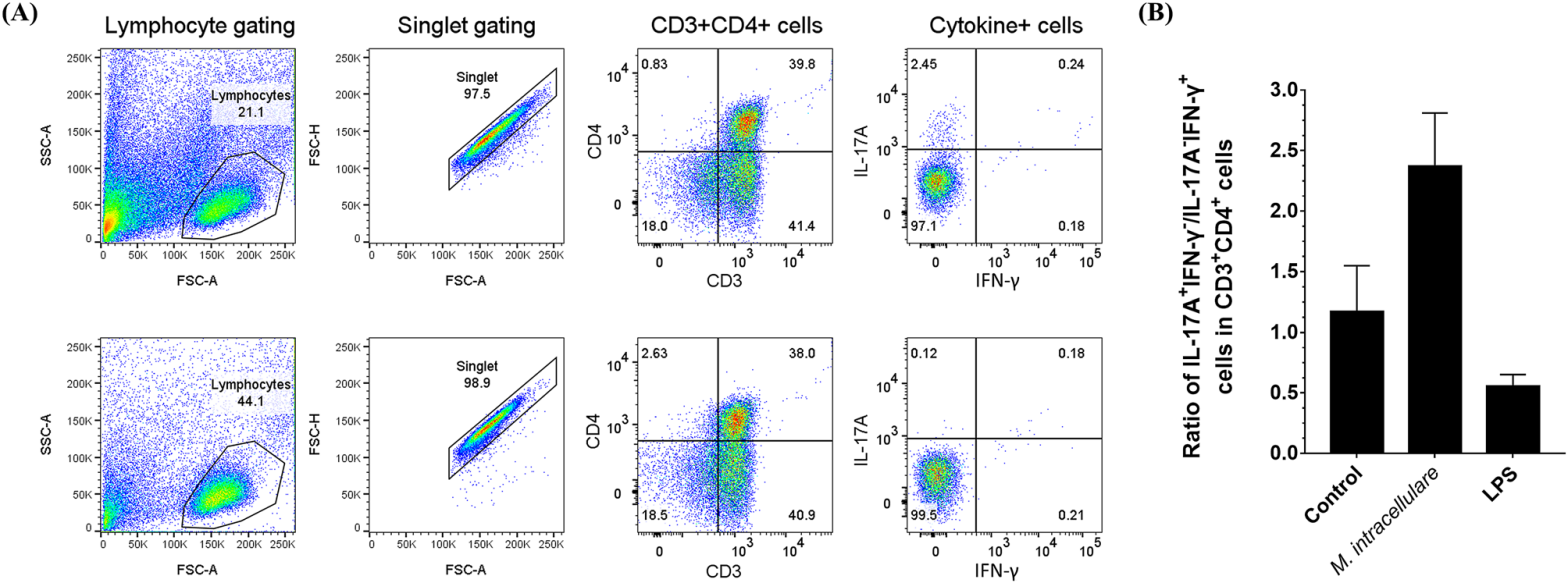
IL-17- and IFN-γ-producing cells among canine CD4^+^ T cells in response to *M. intracellulare*. (**A**) Example of the flow cytometry gating strategy of IL-17- or IFN-γ- producing canine CD4^+^ T cells co-cultured with *M. intracellulare*-infected MDMs for 5 days (upper row) or noninfected MDMs (lower row). (**B**) Bar graph presenting the ratio of the proportion of IL-17-producing CD4^+^ T cells to IFN-γ-producing CD4^+^ T cells. Monocyte-depleted PBMCs were co-cultured with noninfected (control), *M. intracellulare*-infected, or LPS-stimulated MDMs. Each bar presents the mean ± SEM from five individuals.

T cell responses according to *M. intracellulare* were identified at a single-cell level using flow cytometry. To confirm the impact of *M. intracellulare* infected MDMs, canine lymphocytes were co-cultured with non-autologous MDMs infected with *M. intracellulare* for five days (Fig. 6). CD4^+^ T cells in the co-cultured cells were analyzed for the Th17 responses (Supplementary Fig. S3). The canine CD4^+^ T cells polarized moderately to IL-17-producing cells by *M. intracellulare*-infected MDMs independent of T cell receptor recognition (Fig. 6A). Next, lymphocytes were co-cultured with the non-autologous MDMs by replacing the antigenic recognition with the anti-CD3 antibody. The canine CD4^+^ T cells were highly polarized to IL-17-producing cells by replacing MHC recognition. The IL-17 expression was confirmed in lymphocytes co-cultured with infected MDMs treated with anti-CD3 antibody compared to the isotype control and in lymphocytes co-cultured with non-infected MDMs treated with anti-CD3 antibody (Fig. 6D). The IL-17 expression was confirmed in the other two individual canine lymphocytes by comparing the percentage and mean fluorescence intensity (Fig. 6B,C). For validating the antigenic specificity of T cell response, lymphocytes were co-cultured with autologous MDMs, which were non-infected or infected with *M. intracellulare*, and treated with LPS (Fig. 7A). The percentage of IL-17A-producing CD4^+^ T cells specific to *M. intracellulare* was higher than those of IFN-γ-producing CD4^+^ T cells (Fig. 7B). The production of IFN-γ from CD4^+^ T cells was hardly observed in the response by *M. intracellulare*-infected MDMs (Figs. 6, 7). Of note, IFN-γ produced by CD4^+^ T cells was confirmed by stimulation with lipopolysaccharide (Supplementary Fig. S4). Overall, *M. intracellulare* activated macrophages to M1-like phenotype, and *M. intracellulare*-infected macrophages polarized CD4^+^ T cells to Th17 cells.

## Discussion

*Mycobacterium avium* complex has been highlighted as an emerging opportunistic intracellular pathogen that is ubiquitous in the environment, often leading to high rates of host–pathogen contact. In the case of dogs, it has been reported that domestic dogs are more susceptible to mycobacteriosis caused by MAC^3^. Therefore, it is important to investigate the MAC infection in dogs not only for treatment and diagnosis but also to prevent potential zoonotic risk. However, the susceptibility and pathogenicity of MAC in dogs have been remained to be revealed because of the difficulty to evaluate the canine immune response in vitro. Therefore, we investigated the canine immune response against *M. intracellulare* infection by co-culture systems of canine T helper cells and MDMs. Our study showed that susceptibility and inflammatory responses to *M. intracellulare* in dogs were regulated by Th17 cells driven by M1-like macrophage activation.

Transcriptome analysis showed that MDMs were activated to the M1-like macrophage state and secreted cytokines to induce Th1/Th17 cell responses to *M. intracellulare* infection. Top 20 canonical pathways showed that M1 macrophage-related pathways were activated, while M2 macrophage-related pathways were negatively modulated. These pathways were modulated by commonly expressed molecules at 6, 24, and 72 h. Among the molecules, chemokines (*CCL3*, *CCL4*, *CCL5*) and cytokines (*IL6*, *IL1B*) are known to induce M1 macrophage polarization in response to mycobacterial infection. *Mycobacterium*-infected macrophages secrete these proinflammatory mediators that play a key role in leukocyte recruitment^20^. These molecules induce protective immune responses upon M1 polarization^21^. *ICAM1* is an adhesion molecule whose expression is increased by proinflammatory cytokines in an NF-κB-dependent manner^22^. *ICAM1* has been reported to regulate macrophage activation by inhibiting M2 polarization^23^. Proinflammatory cytokines also induce *CSF2*, which activates the expression of cytokines and chemokines to enhance macrophage differentiation to the M1 state^24,25^. *TREM1* increases *IL-1β* production through M1 macrophages as a key player in protective innate immunity during MAC infection^26,27^. *CD40* is a representative marker of M1 macrophages involved in antibody isotype switching and vascular inflammation^28^. Conversely, the M2 macrophage marker *CD36* was downregulated during *M. intracellulare* infection. *CD36* regulates the nuclear receptor *PPARγ* as a class B scavenger receptor for the endocytosis of triacylglycerol-rich lipoprotein particles^29^. *PPARγ* enhances the M2 macrophage response, and lipid modification and repair are fundamental properties of M2 macrophage function^30^.

After identifying the pattern of the immune response by the top 20 canonical pathways, the significantly expressed pathways were analyzed in relation to macrophage activation. The activation states of these pathways showed that most M1 activation-related pathways were activated and they are known to be involved in proinflammatory and protective immune responses against bacterial invasion. On the other hand, M2 activation pathways were negatively modulated and these pathways are related to modulating the nuclear receptor known to enhance M2 activation^29,30^. However, some of the identified pathways were not consistent with the M1 activation state. Especially, apoptosis-related pathways were negatively regulated during the infection. Nevertheless, these states might help explain the reported properties of MAC after macrophage invasion. MAC members are known to replicate within macrophages by preventing apoptotic cell death induced by extrinsic or intrinsic apoptotic pathways^31–33^. Our results also showed that the amount of *M. intracellulare* was increased after 6 days of invasion. However, further study needs to identify the apoptosis of canine macrophages is inhibited by intracellular *M. intracellulare*. These results differ from the classic M1 phenotype known to have a bactericidal function but are consistent with the MAC characteristics after macrophage invasion. Gene expression profiling of macrophage polarization markers showed that only M1 macrophage-related genes were significantly expressed. These results reveal that canine MDMs differentiate into M1-like macrophages during *M. intracellulare* infection, although the activation states of pathways involved in macrophage polarization are distinct from the classic M1 and M2 macrophage phenotypes^8,9^. However, recent studies have reported that macrophages polarize into a unique macrophage population during mycobacterial infection^11^. In particular, Tatano et al. reported that *M. intracellulare* induces a novel type of macrophage population that downregulates the Th1/Th2 cell response, while driving Th17 polarization^12^.

Our results also indicated that the canine MDMs secreted molecules to induce Th17 polarization in response to *M. intracellulare* infection. The top 20 canonical pathways showed that the activated pathways were related to the Th1/Th17 responses. Among the upregulated molecules of that pathways, chemokines (*CCL3*, *CCL4*, *CCL5*, *CCL7*) and cytokines (*IL-1B*, *IL-6*, *CSF2*), were responsible for mediation of the innate immune response in Th1 and Th17 cells during mycobacterial infection^34–36^. However, most of the upregulated molecules were associated with inducing pathogenic Th17 cells during mycobacterial infection. In particular, *CCL3*, *CCL4*, and *CCL5* were reported as pathogenic Th17-signature markers during mycobacterial infection^37,38^. *CSF2* (*GM-CSF*) is also known to contribute to the pathogenicity of Th17 cells^39^. The chemokine *CXCL8* and the adhesion molecule *ICAM1* are directly associated with the Th17 response, which induces the maturation and activation of neutrophils during mycobacterial infection^35,40,41^. In addition, the alarmin *S100A8*, known to contribute to neutrophil accumulation during chronic mycobacterial infection, stimulates *IL-6* to promote Th17 differentiation^42,43^. Moreover, *PTGS2* (*COX-2*), which is involved in *PGE2* production after mycobacterial infection, causes a dysfunctional immune response that favors the survival and replication of mycobacteria by inducing the development of Th17 cells^44,45^. In addition, negatively modulated pathways (LXR/RXR Activation, PPARα/RXRα Activation, PPAR Signaling) were also related to the induction of the Th17 cell response. Nuclear receptor peroxisome proliferator-activated receptors, which primarily regulate these pathways, are known to be major negative regulators of Th17 differentiation^46,47^. These results were also confirmed by both of the ‘Th1 Pathway’ and ‘Th17 Activation Pathway’. Only the Th17 Activation Pathway was activated throughout the infection. In addition, gene expression profiling showed that the genes *IL-23, IL-1β*, and *IL-6*, which induce Th17 cell polarization, were significantly upregulated during *M. intracellulare* infection. In contrast, only *TNF-α* was significantly expressed among molecules related to Th1 cell polarization.

To identify the Th17 cell response against *M. intracellulare* infection, gene expression was quantified in canine lymphocytes. The result showed that Th17-related genes were significantly upregulated in lymphocytes, similar to the finding in macrophages. Moreover, ELISA indicated that IL-17A and IL-1β were produced at higher levels than other cytokines. Furthermore, IL-17A-expressing CD4^+^ T cells were higher expressed in comparison to IFN-γ after *M. intracellulare* infection. The results showed that canine MDMs infected with *M. intracellulare* provide an environment that may favor Th17 responses. Our results were similar to those of studies that showed the production of IL-17 after infection with the *Mycobacterium avium* complex. A study by Kannan et al. showed that *M. avium* infection induced strong induction of IL-17-producing T cells in mice^48^. In particular, a study by Matsuyama et al. reported that the Th17 response was shown to have pathological effects by increasing the susceptibility to systemic *M. avium* infection under Th1-diminished conditions^15^. Th17 cell development is also known to lead to excessive neutrophilic pulmonary inflammation, which plays a pivotal role in granuloma formation and mycobacterial disease development^18,19^. These results will help explain why most reported cases in dogs showed granulomatous inflammation after mycobacterial disease progression induced by MAC members^3,49–52^.

Due to the increase of mycobacterial diseases caused by MAC members in dogs, the need to elucidate the canine immune response has been increased. In this study, the host immune response to *M. intracellulare* was investigated using in vitro canine model with autologous macrophages and lymphocytes. Our results suggested that *M. intracellulare* induces M1-like macrophage polarization that provides a cytokine milieu favoring Th17 response in dogs. This study might open the new horizon in the understanding of MAC infection, especially immune response in dogs and help to control the potential public health risk of zoonosis between dogs and humans.

## Methods

### Ethics statement

This study was conducted in accordance with the regulations of the Seoul National University Institutional Biosafety Committee (protocol: SNUIBC-R190906-1). Blood samples were obtained in accordance with the Guide for the Care and Use of Laboratory Animals and the Animal Welfare Act, and protocols were approved by the Institutional Animal Care and Use Committee (IACUC) at GENIA (IACUC number; ORIENT-IACUC-20003).

### Bacterial strains and cultivation

*Mycobacterium intracellulare* ATCC13950 was kindly provided by Prof. MK Shin from the College of Medicine, Gyeongsang National University, Republic of Korea. *M. intracellulare* was cultured on Middlebrook 7H10 agar (BD Biosciences, CA, USA) containing 0.1% casitone (BD Biosciences) and Middlebrook 7H9 broth supplemented with 0.04% casitone after 7 days. The infection amount was standardized by using the cells at an optical density of 0.244 at 660 nm (4.9 × 10^7^ CFU/ml). The optical density was measured after vigorous vortexing for 30 s to remove clumps.

### Blood cell isolation

Blood samples from 10 healthy beagle dogs were obtained from the animal facility of the 2nd Research Center, GENIA (Eumseong-gun, Chungchengbuk-do, Korea). Professional veterinarians collected whole blood from unanesthetized dogs after clinical, biochemical, and haematological evaluation. The 1-year-old male beagle dogs were routinely vaccinated against Bordetella, rabies, distemper, canine adenovirus type 2, parvovirus, parainfluenza, and leptospirosis. Whole blood was diluted 1:1 in RPMI 1640 medium (Gibco, NY, USA) containing 20% inactivated fetal bovine serum (FBS; Gibco). Peripheral blood mononuclear cells were collected via density gradient centrifugation with Leucoseptube (Greiner Bio-One, Kremsmünster, Austria) containing 1.077 g/ml Ficoll-Paque PLUS (GE Healthcare, Uppsala, Sweden). The PBMCs were suspended in Dulbecco’s phosphate-buffered saline (DPBS) containing 5% FBS, 1% penicillin/streptomycin, and heparin (2000 U/ml) and centrifuged at 400×*g* for 15 min. The cells were filtered with a 100 µm cell strainer (Falcon, Corning, Durham, USA) after washing with DPBS and centrifuged at 300×*g* for 10 min. After repeating the wash step twice, the PBMCs were resuspended in RPMI 1640 medium containing 20% FBS and 1% penicillin/streptomycin for each individual.

### Generation of canine monocyte-derived macrophages

Monocytes were positively isolated using anti-CD14 magnetic microbeads (Miltenyi Biotec Inc., Bergisch Gladbach, Germany). The purity of isolated monocytes was above 99% and analyzed using the anti-CD14 antibody (TÜK4; Bio-Rad) by flow cytometry (FACS Canto II, BD Biosciences, San Diego, CA, USA) (Supplementary Fig. S5). The monocyte was cultured with a RPMI 1640 medium containing 10% heat-inactivated FBS and 1% penicillin/streptomycin and added with 50 ng/ml recombinant human M-CSF (Peprotech Inc., Rocky Hill, HJ, USA) every 2 days for 5 days^53,54^. The morphology was confirmed as the macrophage after 5 days.

### Co-culture of canine lymphocytes and autologous/non-autologous monocyte-derived macrophages infected with *M. intracellulare*

The canine MDMs were seeded at a density of 1 × 10^6^ cells/well into 6-well plates containing RPMI 1640 medium supplemented with 10% FBS. The MDM was infected with *M. intracellulare* at a multiplicity of infection (MOI) of 1 for 24 h because it is sufficient time for processing the antigen. The MDM as control was stimulated with 1 µg/ml of lipopolysaccharide (LPS; Sigma-Aldrich, St Louis, MO, USA) or was treated with DPBS. After 24 h of infection, the MDMs were washed and co-cultured with monocyte-depleted PBMCs at responders: stimulators (MDMs) ratio of 10:1. The autologous MDMs and monocyte-depleted PBMCs were co-cultured for 0–144 h to characterize the *M. intracellulare*–specific response and the effect of MDM–derived cytokines on T cells. The co-cultured cells for 5 days were confirmed as CD3^+^ T lymphocytes by flow cytometry.

The anti-dog CD3 antibody (clone CA17.2A12) effectively activated canine lymphocytes as the anti-human CD3 antibody (clone OKT3) does^55,56^. The monocyte-depleted PBMCs were co-cultured with *M. intracellulare*-infected non-autologous MDMs with or without anti-CD3 antibodies (clone CA17.2A12). The monocyte-depleted PBMCs were also co-cultured with non-autologous MDMs with the anti-CD3 antibody. After 5 days of co-cultures, the lymphocytes were restimulated with anti-CD3 antibodies. The activated lymphocytes were analyzed by surface staining and intracellular cytokine staining assay. The data were visualized using FlowJo v10 software (BD Biosciences; https://www.flowjo.com/).

### RNA expression, library preparation, and sequencing

Total RNA was isolated from canine MDMs after *M. intracellulare* infection for 0, 6, 24, and 72 h using an RNeasy Mini Kit (Qiagen, Hilden, Germany). Noninfected MDMs were used as a negative control. Eighty RNA samples were extracted from ten autologous MDMs of both infection and non-infection groups at four-time points. Each five RNA samples were pooled and a total of 16 cDNA libraries was sequenced. The RNA quality was identified by an Agilent 2100 bioanalyzer using an RNA 6000 Nano Chip (Agilent Technologies, Amstelveen, The Netherlands), and RNA was quantified on an ND-2000 spectrophotometer (Thermo Inc., Waltham, MA, USA). The RNA library was constructed with a QuantSeq 3′ mRNA-Seq Library Prep Kit (Lexogen, Inc., Vienna, Austria) according to the manufacturer’s instructions. In brief, the 5′ end of an Illumina-compatible sequence containing an oligo-dT primer was hybridized to the total RNA. After reverse transcription, the RNA template was degraded to synthesize the second strand using an Illumina-compatible linker sequence containing a random primer. To remove all reaction components, the double-stranded library was purified by magnetic beads. The library was amplified with complete adapter sequences and purified from PCR components. High-throughput sequencing was performed with single-end 75-bp sequencing using a NextSeq 500 (Illumina Inc., CA, USA). The RNA-Seq data were deposited into the NCBI Gene Expression Omnibus (GEO) database under accession number GSE158425.

### RNA-sequencing read alignment and data analysis

QuantSeq 3′ mRNA-Seq reads were mapped to canFam 3 using Bowtie2^57^. Both the genome assembly sequence and the representative transcript sequences generated Bowtie2 indices. After aligning the transcriptome, transcripts were assembled to identify differentially expressed genes. DEGs were identified based on counts from unique and multiple alignments determined by Bedtools^58^. The read counts were processed based on the quantile normalization method using EdgeR within the R package with Bioconductor^59^. The DEGs were analyzed by Ingenuity Pathway Analysis (IPA; Qiagen Inc., https://www.qiagenbioinformatics.com/products/ingenuitypathway-analysis)^60^. Each group was subjected to core and comparative analyzes, and signalling pathways were predicted by the molecule activity predictor in IPA.

### Quantification of gene expression

Total RNA was extracted from both canine MDMs infected with *M. intracellulare* for 0–72 h and lymphocytes co-cultured with *M. intracellulare-*infected MDMs for 0–144 h using an RNeasy Mini Kit (Qiagen, Hilden, Germany). Noninfected MDMs and lymphocytes were used as controls. Gene expression was quantified by RT-qPCR to identify the activation of macrophages and T lymphocytes. In addition, RT-qPCR was performed with randomly selected genes to validate the RNA-Seq data (Supplementary Fig. S6). cDNA was synthesized using a High-Capacity cDNA Reverse Transcription Kit (Applied Biosystems, Foster City, CA, USA), and RT-qPCR was performed with Real-Time qPCR 2 × Master Mix (SYBR Green, Elpisbiotech, Daejeon, Republic of Korea) on a Rotor-Gene Q real-time PCR cycler (Qiagen, Hilden, Germany). cDNA was amplified using the canine primers shown in Supplementary Table S6. The amplification conditions were as follows: 95 °C for 3 min and 45 cycles of 95 °C for 3 s and 30 s at 60 °C. A melting curve was constructed for the detection of nonspecific product formation, and the gene expression levels were determined via the 2^−ΔΔCt^ method using glyceraldehyde-3-phosphate dehydrogenase (GAPDH) as the reference gene. The fold change was determined based on the relative gene expression level compared to the control.

### Quantification of cytokines

Lymphocytes were co-cultured at 1 × 10^7^ cells/well with *M. intracellulare*-infected MDMs in 6-well plates for 0–144 h. The cytokine concentrations in the supernatants were determined by ELISA. Canine IL-17A, IL-1β, IL-12, and IL-4 were detected by DuoSet (R&D Systems, Minneapolis, MN, USA) and IFN-γ, IL-6, IL-10, and TNF-α were detected by Quantikine ELISA kits (R&D Systems) according to the manufacturer’s instructions.

### Intracellular cytokine staining assay

Lymphocytes were co-cultured with *M. intracellulare*-infected MDMs to analyze T helper cell polarization. After 5 days of incubation, the cells were incubated with 2 µg/ml brefeldin A (Sigma) for 4 h. The cells were stained with the LIVE/DEAD Fixable Aqua Dead Cell Stain Kit (Invitrogen) and subsequently stained with FITC-anti-CD3 (clone CA17.2A12) and RPE-anti-CD4 (clone YKIX302.9) (both from Bio-Rad Laboratories; Hercules, CA, USA) for 20 min at RT in the dark. After surface staining, the cells were washed with PBS containing 2% FBS and fixed and permeabilized (BD Biosciences, San Diego, CA, USA) for 30 min at 4 °C. The cells were washed twice with BD Perm/Wash buffer (BD Biosciences) and stained with eFluor 450-anti-IL-17A (clone eBio17B7; eBioscience, San Diego, CA, USA) and Alexa Flour 647-anti-IFN-γ (clone CC302; Bio-Rad) for 30 min at 4 °C. The eFluor 450-rat IgG2a kappa Isotype Control (clone eBR2a; eBioscience, San Diego, CA, USA) used for isotype control of the anti-IL-17A antibody. Single-stained samples for each antibody were used to compensate for the fluorescence spillover. The data were analyzed using FlowJo v10 software (BD Biosciences).

### Intracellular survival and replication

The bacterial invasion assay was conducted as previously described with slight modification^61,62^. MDMs were infected for 2 h with *M. intracellulare* at an MOI of 1. Cells were washed with DPBS after centrifugation at 400×*g* for 5 min. Extracellular bacteria were eliminated by treatment with amikacin at a concentration of 200 µg/ml for 2 h. Following incubation, the cells were lysed with 1% Triton X-100 (Sigma-Aldrich, MO, USA). The viable intracellular bacteria were enumerated by serial dilution and plating onto Middlebrook 7H10 agar containing 0.1% casitone after vigorous vortexing for 30 s.

### Statistical analysis

Statistical significance was analyzed by an unpaired t-test with Welch’s correction using GraphPad Prism version 7.00 (Windows, GraphPad Software, La Jolla California USA, http://www.graphpad.com) for comparisons between the *M. intracellulare*-infected and non-infected groups. Significant differences were determined at *p* < 0.05. Fold change is represented by the mean ratio of gene expression in *M. intracellulare*-infected cells/noninfected cells.

## Supplementary Information

Supplementary Figures.

Supplementary Tables.

## Data Availability

Raw files and normalized datasets are available from the Gene Expression Omnibus (GEO) https://www.ncbi.nlm.nih.gov/geo/query/acc.cgi?acc=GSE158425 under the accession number GSE158425.
