# Supplementary material for: Mycobacterium intracellulare induces a Th17 immune response via M1-like macrophage polarization in canine peripheral blood mononuclear cells
Source: Sci Rep. 2022 Jul 12;12:11818. doi: 10.1038/s41598-022-16117-2 (PMC9276657; doi:10.1038/s41598-022-16117-2)
Supplement: Supplementary file 1 — Supplementary Figures. [file 41598_2022_16117_MOESM1_ESM.docx]

**Supplemental Material**


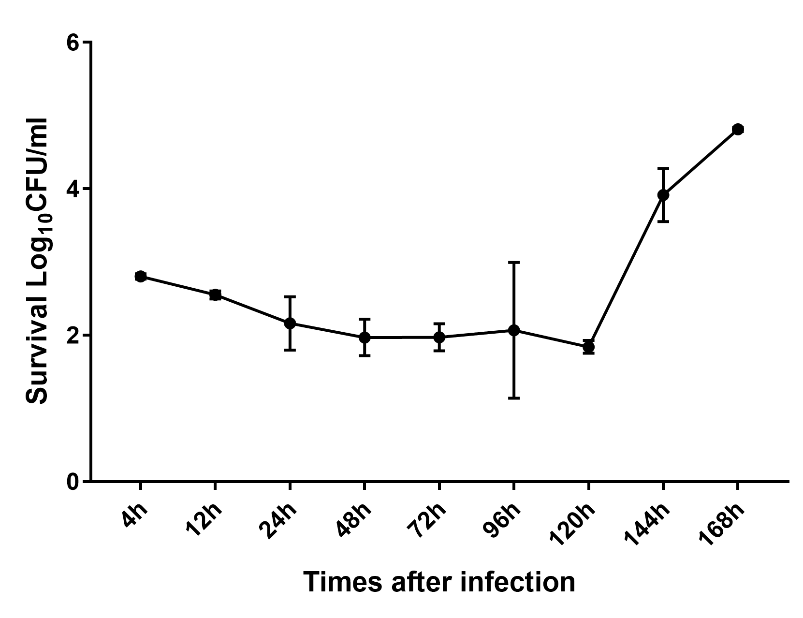


Supplementary figure S1. Intracellular survival and replication of *M. intracellulare* in canine monocyte-derived macrophages. Intracellular survival of *M. intracellulare* was examined in canine MDMs for 7 days. Data are shown as mean $\pm$ SEM of three independent experiments.

**
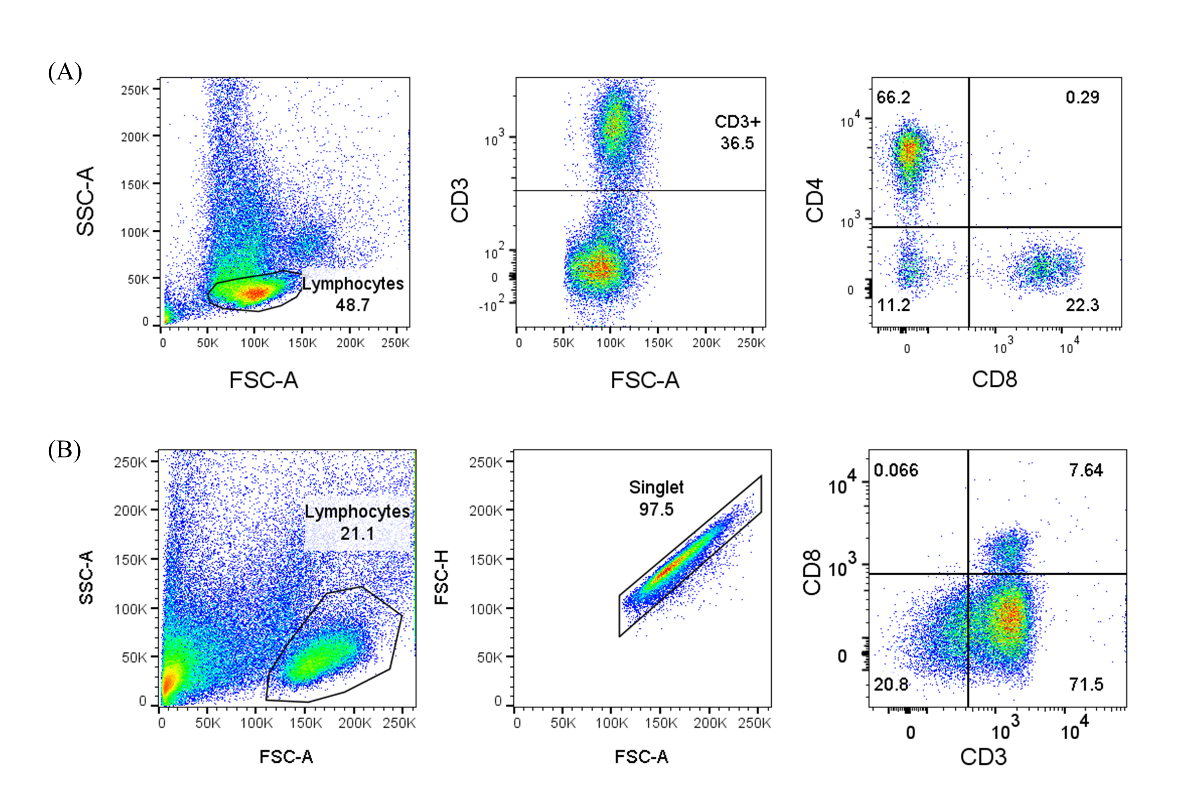
**

Supplementary figure S2. The proportion of T cells in PBMCs (A) and co-cultured cells (B). (A) The proportion of CD4^+^ T cells and CD8^+^ T cells (right) were analyzed from CD3^+^ T cells (middle) in lymphocytes (left). (B) CD8^+^ T cells (right) were analyzed from singlet lymphocytes (middle and left) co-cultured with for six days with *M. intracellulare*-infected MDMs.


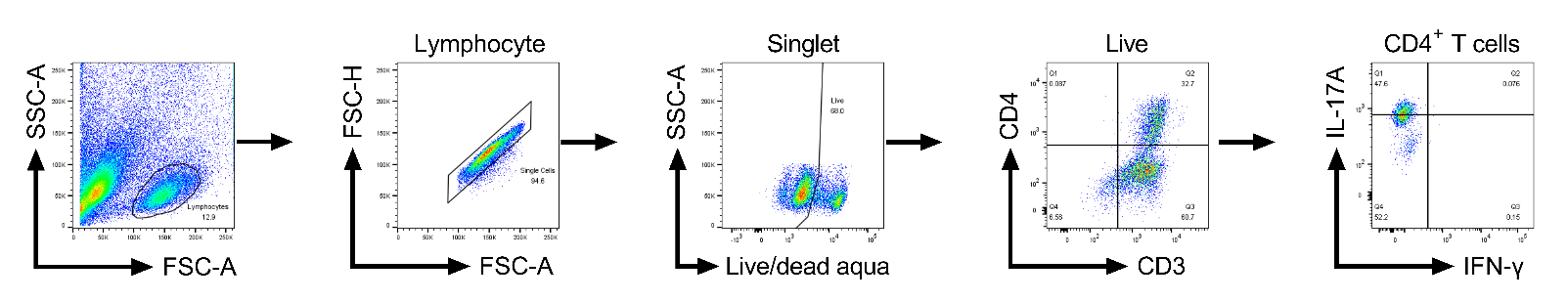


Supplementary figure S3. Representative gating strategy for the flow cytometric analysis of IL-17-producing CD4 T cells. Numbers in plots indicate the percent cells in respective gates.


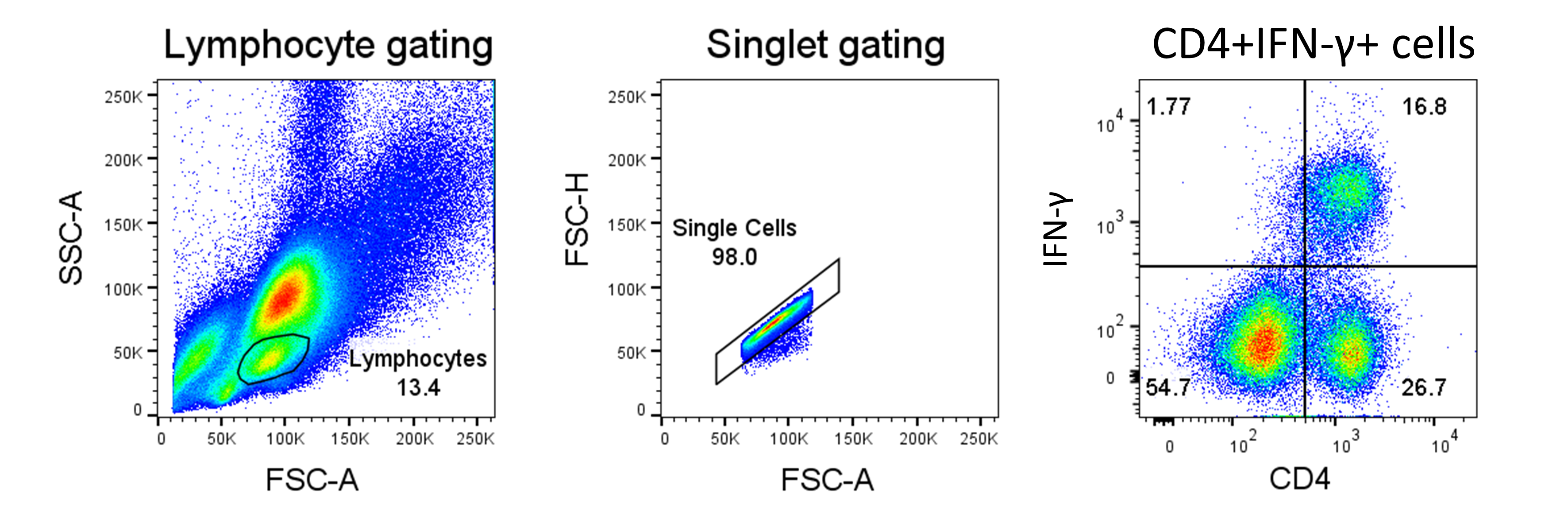


Supplementary figure S4. IFN-γ producing CD4^+^ T cells from lymphocytes stimulated with LPS. Lymphocytes were stimulated with 1ug/ml of LPS overnight. IFN-γ production in canine CD4^+^ cells were confirmed by intracellular cytokine staining.


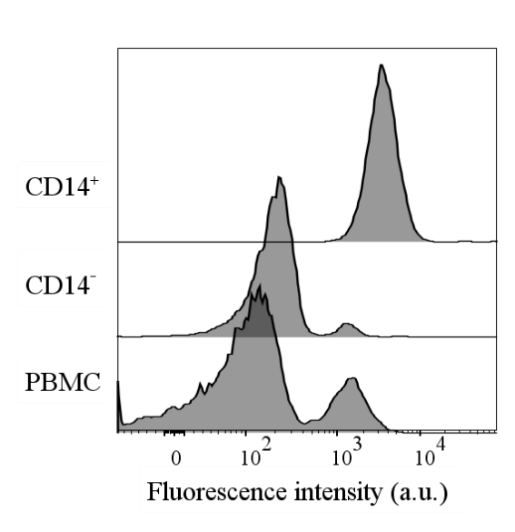


Supplementary figure S5. The purity of isolated monocytes. The proportion of CD14^+^ cells in positively isolated CD14^+^ cells (upper), CD14 negative fraction (middle), and PBMCs (lower) by flow cytometry.

**
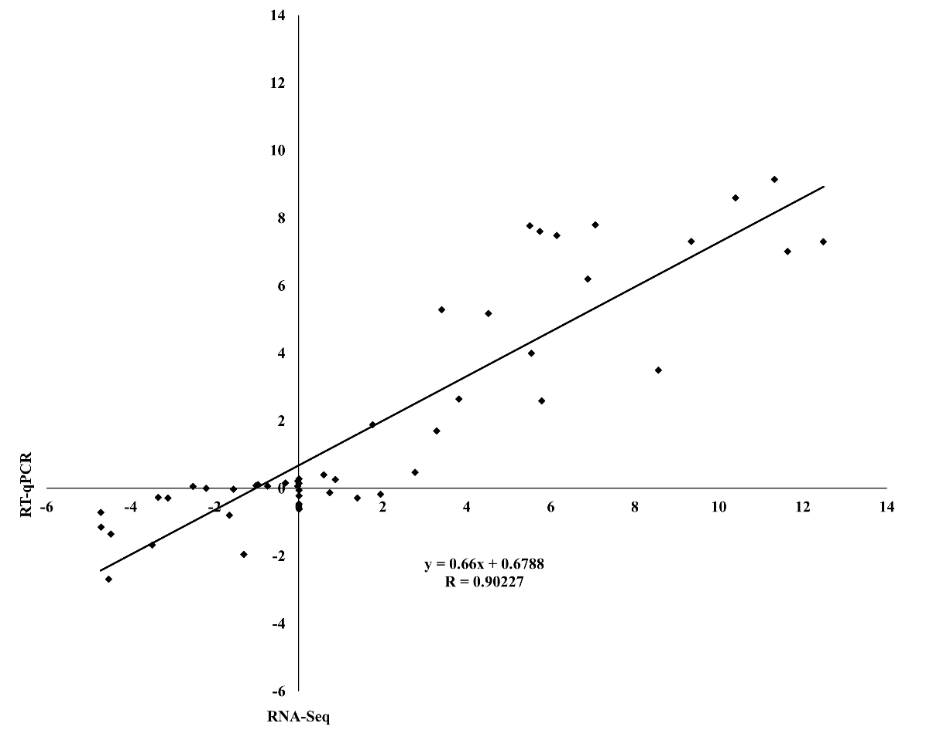
**

Supplementary figure S6. Validation of gene expression by RNA-Seq and quantitative real-time PCR. The relative expression levels were compared to those observed in the control cells to determine the fold changes in expression for each gene.
